# Obesity-induced activation of NADPH oxidase 2 prolongs cardiac repolarization via inhibiting K^+^ currents

**DOI:** 10.1371/journal.pone.0316701

**Published:** 2024-12-31

**Authors:** Bin Li, Yating Chen, Maoxiang Zhao, Zhijie Chen, Zhuhui Lin, Jie Liu, Xueping Wang, Jiancheng Zhang, Yang Li

**Affiliations:** 1 Chinese PLA Medical School, Chinese PLA General Hospital, Beijing, China; 2 The Eighth Medical Center of PLA General Hospital, Beijing, China; 3 Senior Department of Cardiology, the Sixth Medical Center of PLA General Hospital, Beijing, China; 4 Department of Cardiology, Fujian Provincial Hospital, Provincial Clinical Medicine College of Fujian Medical University, Fuzhou, China; 5 Medical Innovation Research Department of PLA General Hospital, Beijing, China; The Open University, UNITED KINGDOM OF GREAT BRITAIN AND NORTHERN IRELAND

## Abstract

Obesity is associated with abnormal repolarization manifested by QT interval prolongation, and oxidative stress is an important link between obesity and arrhythmias. However, the underlying electrophysiological and molecular mechanisms remain unclear. The aim of this study is to evaluate the role of obesity in potassium current in ventricular myocytes and the potential mechanism of NADPH oxidase 2 (Nox2). We investigated the effect of Nox2 on cardiac repolarization without compromising its expression and function in other systems using mice with conditional cardiac-specific deletions of Nox2 (knockout [KO]). Wild-type, KO, and Flox littermate mice were randomized to either the control or high-fat diet (HFD) groups. Surface electrocardiograms were recorded to analyze repolarization in vivo. Whole-cell patch-clamp techniques were used to evaluate the electrophysiological phenotype of isolated myocytes in vitro. Western blotting was performed to assess protein expression levels. Compared with the control mice, the HFD group had a prolonged QTc. The consequences of an HFD were not attributed to delayed rectifier K^+^ and inward-rectifier K^+^ currents but were associated with reduced peak outward K_V_ and fast transient outward K^+^ currents. Downregulated expression of K_V_4.2 and KChIP2, comprising functional I_to_ channel pore-forming (α) and accessory (β) subunits, was detected in HFD mice. Nox2-KO reversed the effect of obesity on I_peak_ and I_to_ amplitude. Our data demonstrate that obesity mediates impaired cardiac repolarization in mice, manifested by QTc at the whole organism level and action potential duration at the cellular level, and correlated with Nox2. The electrophysiological and molecular aspects of this phenomenon were mediated by repolarizing outward K^+^ currents.

## Introduction

The occurrence of obesity has doubled in 73 countries between 1980 and 2015 [1]. Obesity and its associated metabolic disorders pose serious threats to public health and account for two-thirds of deaths related to cardiovascular diseases [1, 2]. Long QT syndrome (LQTS), characterized by a prolonged QT interval on the electrocardiogram (ECG), is associated with an extended ventricular action potential duration (APD) [3]. Acquired LQTS is more common than its congenital form [4]. Typical arrhythmias in patients with acquired LQTS include torsades de pointes (TdP), a rapid heart rhythm that can self-terminate or progress to ventricular fibrillation (VF) [5, 6]. Obesity is associated with a longer QTc interval [5, 6], which is an independent risk factor for cardiac arrhythmias. Following bariatric surgery, the QTc interval significantly decreases, indicating a causal relationship rather than a mere correlation [6, 7]. The global trend of obesity highlights the substantial impact it has on compromised cardiac repolarization. However, the underlying electrophysiological and molecular mechanisms of repolarization abnormalities in obesity are not completely understood.

A decrease in outward K^+^ currentsresults in an increased APD and a prolonged QT interval. Some studies have investigated the effect of a high-fat diet (HFD) on ventricular K^+^ channels [8–10]. However, either direct electrophysiology testing was not conducted or the results were limited due to the use of computer modeling.

Numerous studies have demonstrated a link between obesity and ventricular oxidation [11–13]. Reactive oxygen species (ROS) in the cardiovascular system are primarily produced by the Nox family [14–16]. Moreover, studies of atrial fibrillation and hyperglycemia have demonstrated oxidation-dependent regulation of K^+^ channels by NADPH oxidase 2 (Nox2) [17–19]. We hypothesized that HFDs would facilitate the regulation of outward K^+^ channels to prolong cardiac repolarization and that obesity would decrease the activity of major cardiac voltage-gated K^+^ channels via enhanced oxidative stress, creating an electrophysiological remodeling substrate for impaired cardiac repolarization. This may derive from the direct or indirect effect of Nox2 on potassium channels. This study aimed to evaluate the role of obesity onpotassium current in ventricular myocytes and the potential mechanism of Nox2; these objectives were clarified.

## Methods

### Animal model

C57BL6J Mice (weight, 20–22 g; age, 7–8 weeks; male; SPF) were obtained from SPF Biotechnology Co., Ltd. (Beijing, China, SCXK2019-0010). The animals were kept in a pathogen-free facility at 23 ± 2°C and 60 ± 5% relative humidity for 12 hours/12 hours of light/dark cycling. Each group had free access to food and water, and the weight of all mice was recorded once a week. The bedding material was replaced daily to keep it dry. The mice enjoyed a standardized living environment.

All procedures were conducted in accordance with the "Guide for the Care and Use of Laboratory Animals" published by the National Institutes of Health in the United States. All study protocols were approved by Chinese PLA General Hospital’s Ethics Committee (2023-X19-64). Animals were deep anesthetized using isoflurane and euthanized by cervical dislocation. To minimize pain and suffering, this rapid method was performed by trained personnel to ensure immediate loss of consciousness and death.

This research was carried out while ensuring animal welfare. Throughout the research process, the health and behavior of the mice were monitored daily for timely resolution of any signs of pain. The experimental plan followed the "3Rs" principle, minimizing the number of animals used and making every effort to reduce animal suffering while maximizing welfare.

Wild-type mice were randomly assigned to either the HFD or control group. Mice in the HFD group were given an HFD with 60% fat content (D12492, Research Diets) for 12 weeks [18]. Meanwhile, mice in the control group were fed a normal diet. A mouse is considered obese when it reaches 33 g in body weight [19]. To further investigate the effects of Nox2 deletion, we generated *Nox2*^*fl/fl*^ αMyHC-Cre (Nox2-knockout [KO]) mice using αMyHC-Cre, which is specific to mouse hearts. Flox and KO mice were fed either a normal diet or a HFD for 12 weeks and were divided into four groups each. See the Supplemental Materials (S1 File) for further details on the generation of KO mice.

### Body compositions of mice

Body composition was assessed using Minispec LF50 MRI (Bruker, Germany). The instrument’s temperature was maintained at about 37.0°C. Tests were performed on awake mice. Fat, lean mass, and free body fluid were measured.

### Histology

To conduct histological analysis, we euthanize the mice under deep anesthesia with isoflurane. Their limbs were fixed on a foam console using a needle, and water was sprayed on their chest with a watering can to minimize hair loss. The chest of each mouse was then cut open along the "V" shape of the xiphoid process. We bluntly separated the pericardial tissue, incised the blood vessels from the base of the heart, promptly remove the heart, and gently rinsed with physiological saline. Fresh heart tissue samples were taken and fixed in 4% paraformaldehyde. After undergoing gradient dehydration, the embedding process commenced, and the tissue samples were cut longitudinally at 5 μ m for hematoxylin and eosin (H&E) staining or Masson’s trichrome staining. The tissue samples were examined under a microscope and analyzed using the Image J program (NIH).

### Echocardiograms

Echocardiography was performed under 2% isoflurane anesthesia at heart rates >400 bpm using a Vevo2100 system with a 30 MHz linear probe (Visualsonics, Canada). Body temperature was maintained between 36.5°C and 37.5°C. Systolic function parameters, including ejection fraction and fractional shortening, were measured in the two-dimensional parasternal short-axis imaging plane of M-mode tracings close to the papillary muscle level.

### Electrocardiographic recordings and analysis

The ECG was recorded for 5 minutes at a rate of 1 kHz using needle electrodes placed subcutaneously in anesthetized mice under isoflurane anesthesia. The PowerLab system (ADInstruments Ltd., Australia) was used to calculate the heart rate. The ECG intervals were measured manually from the recordings with minimal artifacts using LabChart 8 software, and the electrocardiographic parameters were calculated. Four successive beats were averaged to obtain intervals, and QTc was analyzed using Eq (1) [20]:

$$QTc=QT/{\left(RR/100\right)}^{1/2}$$ (1)

### Cardiomyocyte isolation and whole-cell patch-clamp recordings

Briefly, a Langendorff perfusion system was used to isolate ventricular myocytes.

The amplifier of MultiClamp 700B (Axon Instruments, USA) was used to measure membrane currents. Digidata 1440A (Axon Instruments) was used as the output, and pCLAMP 10.7 was used as the software control. A detailed description is provided in the Supplemental Methods (S1 File).

### Western blotting

Frozen radioimmunoprecipitation assay containing trypsin inhibitors was used to lyse each sample and phenmethylsulfonyl fluoride (1 mM). The LV homogenates were separated using SDS-PAGE. Anti-K_V_4.2 (APC-023), anti-KChIP2 (APC-142), anti-K_ir_2.1 (APC-026), and anti-K_V_1.5 (APC-004, Alomone); anti-Nox2 (Proteintech); and anti-tubulin (Immunoway) were used. The signal intensity was measured by ImageJ (NIH) normalized to β-tubulin.

### Statistical analysis

Data are presented as means ± SEMs. Cell and animal numbers are shown in the figure legends and tables. Differences between the two groups were analyzed using a two-tailed Student’s t-test, and comparisons of multiple groups were performed using either one-way or two-way ANOVA, followed by a post-hoc Tukey’s test. Statistical significance was set at *p*<0.05. Statistical software SPSS 20.0, Origin Pro 8, LabChart 8, and Graph Pad Prism 8.0 were used to conduct the analysis.

## Results

### Prolonged QT intervals in HFD mice

**Fig 1:**
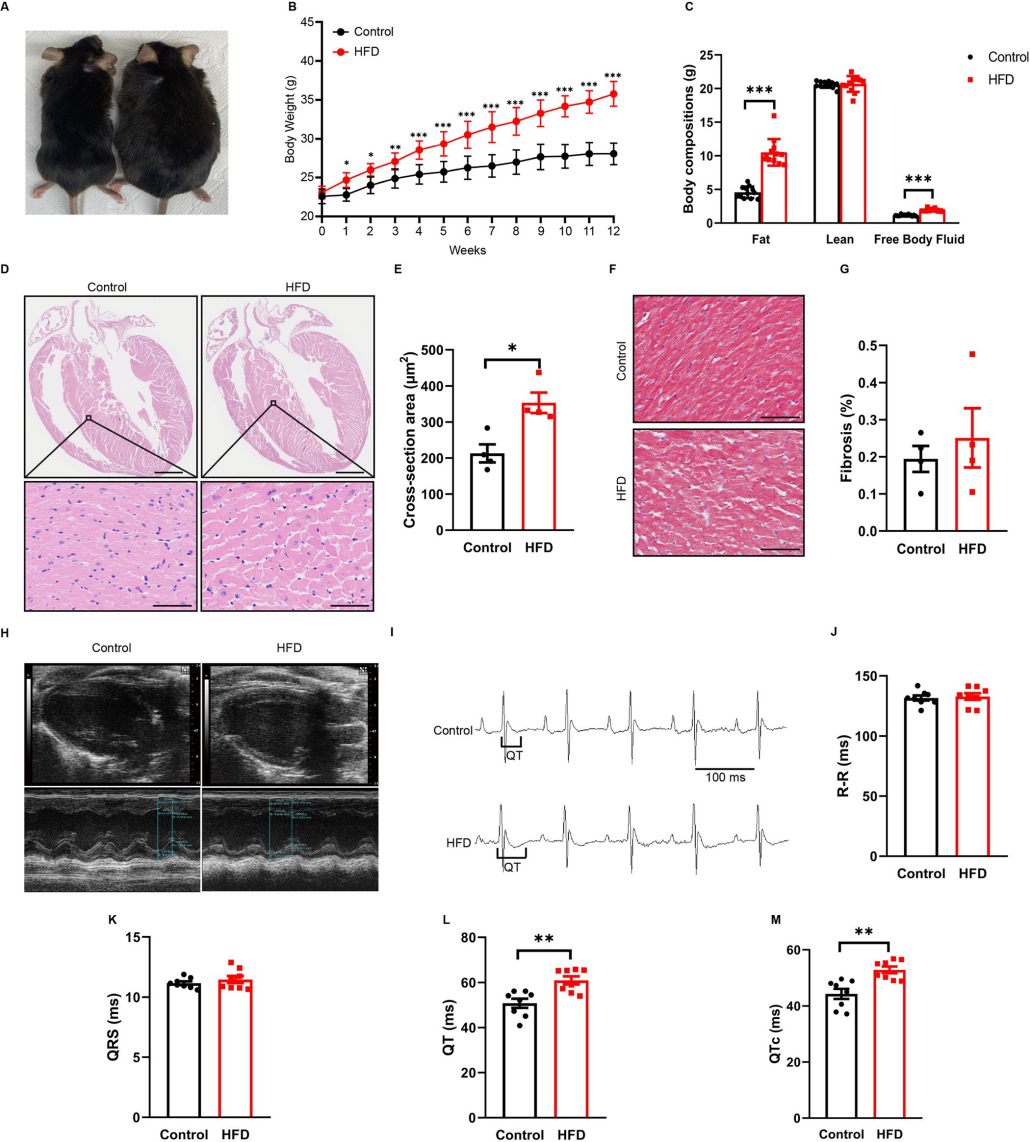
Basic information of two groups of wild-type mice. A: Photos visually display the obesity phenotype of mice. B: Body weights in control and HFD wild-type mice. C: Fat weight, lean weight, and free body fluid content among the two groups. D: Representative whole heart and left ventricle section stained with hematoxylin and eosin from control and HFD wild-type mice. Scale Bars, 500 μm (top panel) and 50 μm (lower panel). E: Quantification of cardiomyocyte cross-section area of the two groups. F: Representative Masson’s Trichrome stained sections from control and HFD wild-type mice. Scale Bars, 50 μm. G: Scatter plot of collagen content in the ventricular area of the two groups. H: Echocardiograms of the two groups. I: ECG recordings from representative mice of the two groups. QT intervals are indicated below the records. Scattered data of R-R interval (J), QRS width (K), QT interval (L) and QTc (M) in the 2 groups at 20weeks. For A–C, n = 12 animals per group; for D–G, n = 4 animals per group; for H–M, n = 8 animals per group. *p<0.05, **p<0.01, ***p<0.001. HFD, high-fat diet.

After 12 weeks of feeding, wild-type HFD mice gained a significantly higher body mass compared to control mice (*p*<0.001) (Fig 1A and 1B). Their fat weight and free body fluid content were also higher (*p*<0.001) (Fig 1C), while lean weight remained unchanged. Under H&E staining, the endocardium, myocardium, and epicardium of the heart tissue were clearly visible within the field of view, and no significant abnormalities were observed in the heart wall or heart chamber. The myocardial cells were clearly demarcated. No significant inflammatory cell infiltration was observed in both the control and HFD groups (Fig 1D). HFD mice showed loose arrangement of myocardial cells and widened intercellular spaces, and cardiomyocyte cross-sectional area increased in HFD (353.1±28.3) compared with the control group (213.0±24.9; *p*<0.05) (Fig 1E). The extent of fibrosis, measured with Masson trichrome staining, did not show significant changes in the two groups (*p*>0.05) (Fig 1F and 1G). To explore the effects of obesity on cardiac structure and function, echocardiography was performed on two groups of mice, and it was found that obesity did not affect LVEF and LVFS (Fig 1H and Table 1). After a surface electrocardiogram test was conducted (Fig 1I), the ECG recordings showed that R-R intervals (Fig 1J) and QRS waves (Fig 1K) showed no significant differences between the two groups. Wild-type mice in the HFD group had longer QT intervals (60.9±1.8ms) than the controls (50.8±2.0 ms, *p*<0.01) (Fig 1L). QTc-Bazett interval differences were also significant, with QTc intervals of 44.3±1.8 ms and 52.8±1.2 ms in the control and HFD groups, respectively (*p*<0.01) (Fig 1M).

**Table 1:** Echocardiographic parameters of HFD mice, compared with those of control mice.

	Control	HFD	*p* value
Heart rate (beats/min)	450±8	439±9	0.76
LVID;d (mm)	3.84±0.07	3.88±0.07	0.66
LVID;s (mm)	2.82±0.09	2.71±0.06	0.34
EF (%)	52.53±2.36	57.87±2.31	0.13
FS (%)	26.61±1.45	30.16±1.60	0.12
LV Vol;d (μl)	63.66±2.93	65.46±2.78	0.66
LV Vol;s (μl)	30.39±2.36	27.42±1.54	0.31

n = 8 animals per group. Data are presented as mean ± SEM. HFD—high-fat diet, LVID;d—left ventricular internal diameter in diastole, LVID;s—left ventricular internal diameter in systole, EF—Ejection fraction, FS—Fractional shortening, LV Vol;d—diastolic volume, LV Vol;s—systolic volume.

### Prolonged APDs in ventricular myocytes of HFD mice

**Fig 2:**
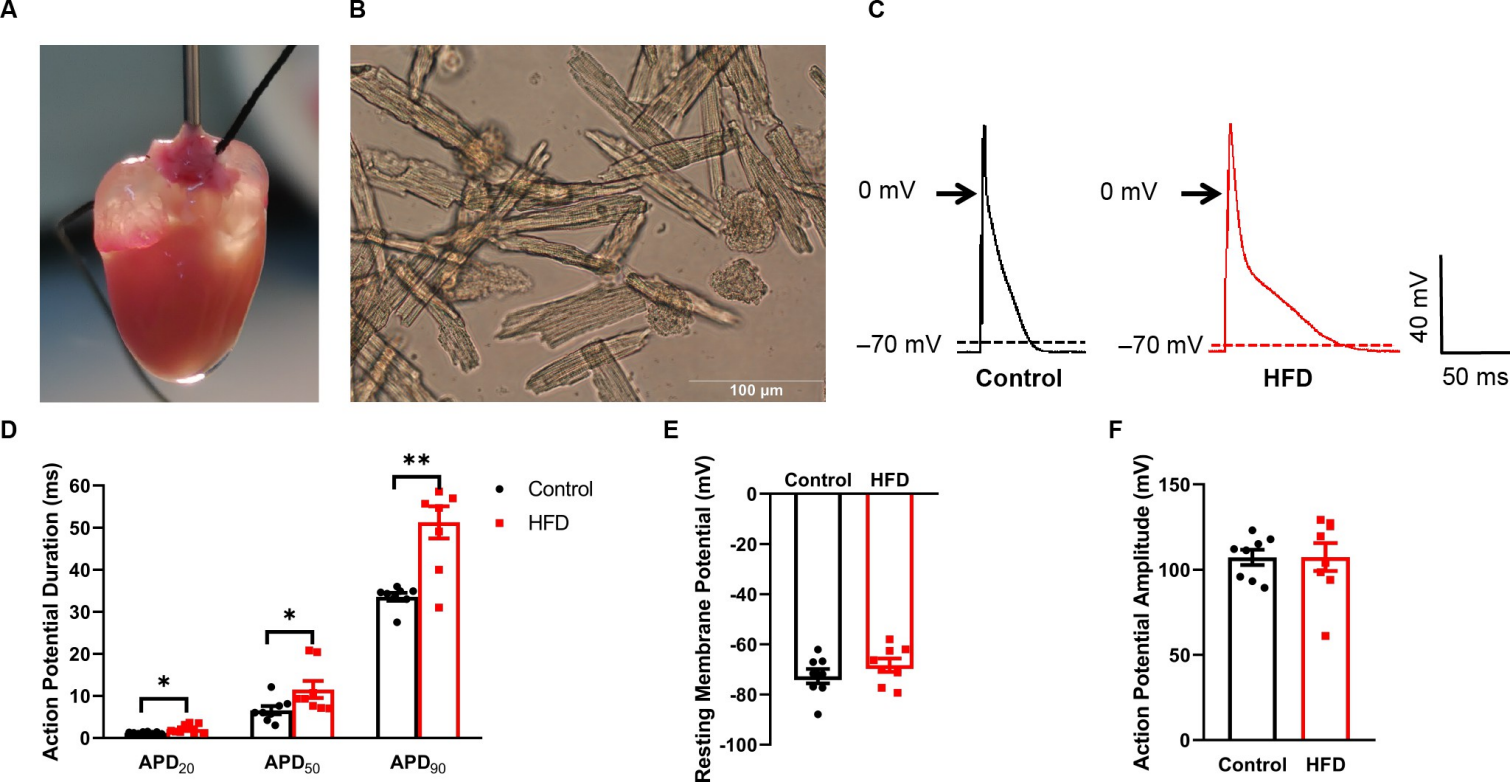
Ventricular action potential duration (APD) is prolonged in HFD mice. A: The mouse heart perfused through retrograde aortic catheterization. B: The digested single ventricular cardiomyocytes. C: Representative AP tracings in control and HFD mice. D: APD at 20%, 50%, and 90% repolarization. Resting membrane potential (RMP) (E) and action potential amplitude (APA) (F) were not different among control and HFD mice. n = 8–11 cells; three hearts per group. *p<0.05, **p<0.01. HFD, high-fat diet.

The mouse heart perfused through retrograde aortic catheterization under a stereomicroscope is shown in Fig 2A, and the digested single ventricular muscle cells are shown in Fig 2B. Using whole-cell patch-clamp electrophysiology, the authors investigated the electrophysiological mechanisms of cardiac repolarization by studying APD_20_, APD_50_, and APD_90_. Representative AP tracings in control and high-fat diet (HFD) mice are shown in Fig 2C. APD_20_, APD_50_, and APD_90_ recorded from the ventricular myocytes of HFD mice were substantially broader (Fig 2D). The data suggest that no significant differences were present in action potential amplitudes (APA) and resting membrane potentials (RMPs) between the two groups (*p*>0.05) (Fig 2E and 2F).

### Attenuated repolarizing currents in ventricular myocytes of HFD mice

**Fig 3:**
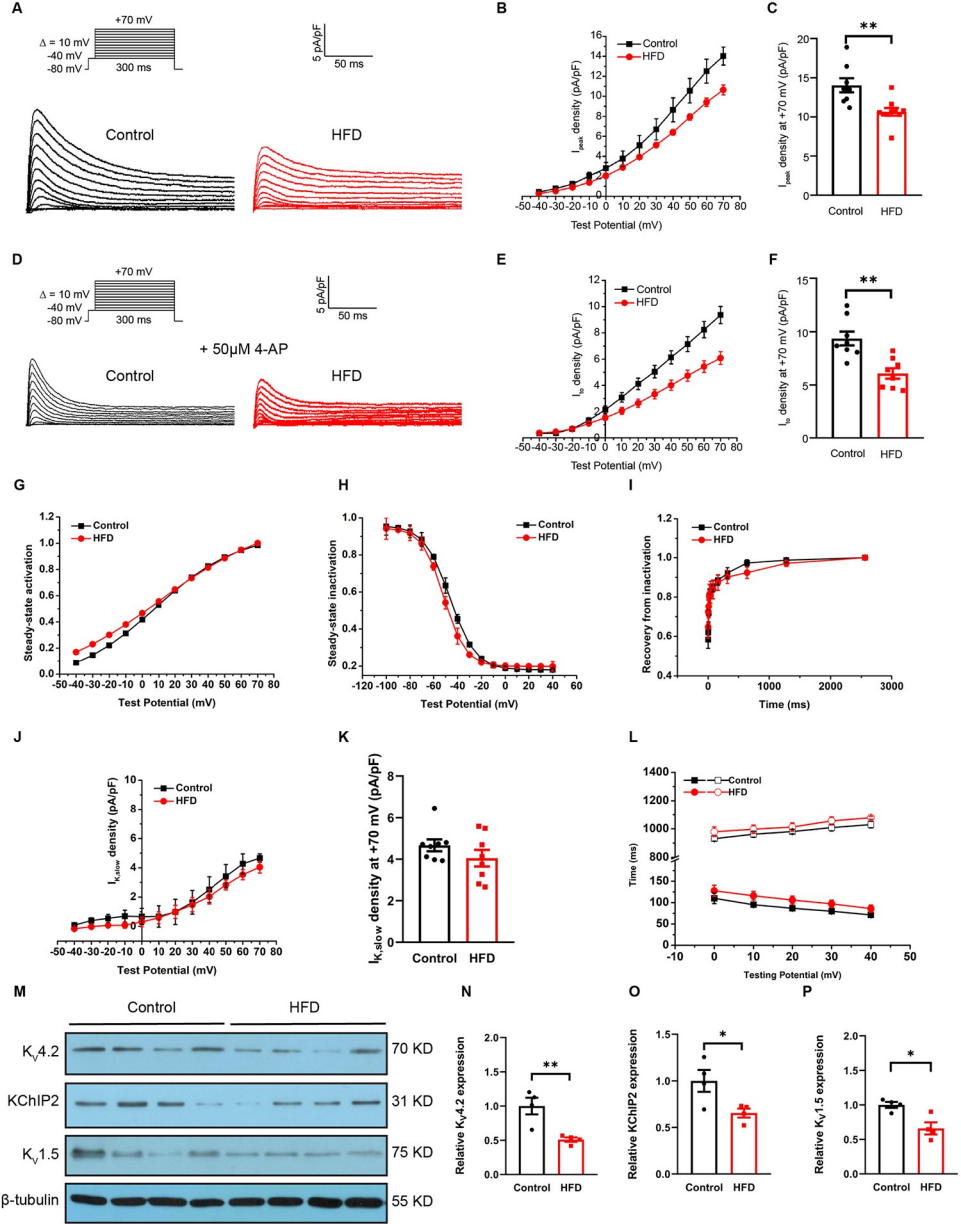
Peak outward K^+^ (I_peak_) and fast transient outward K^+^ (I_to_) currents were reduced in HFD mice. A: Outward K^+^ currents recorded from control (black) and HFD (red) mouse ventricular myocytes were evoked during 300-ms depolarizing voltage steps. B: Voltage relationship (I-V curves) in control and HFD mice. C: I_peak_ at +70 mV in control and HFD mice. D: Representative I_to_ recorded from ventricular myocytes of control (black) and HFD (red) mice. E: Voltage relationship (I-V curves) in the two groups. F: I_to_ at +70 mV in the two groups. G, H and I: Steady-state activation (SSA), steady-state inactivation (SSI), and recovery from inactivation (RFI) curves of I_to_. J: Voltage relationship (I-V curves) of I_K,slow_ in the two groups. K: I_K,slow_ at +70 mV in the two groups. L: Analyzed fast (lower series) and slow (upper series) inactivation time constants (τ values) from currents. M: Representative Western blots of K_V_4.2, KChIP2 and K_V_1.5. N: Quantification of K_V_4.2. O: Quantification of KChIP2. P: Quantification of K_V_1.5. for A-L, n = 8 cells; three or four hearts per group. for M-P, n = 4 animals per group. *p<0.05, **p<0.01.

The hypothesis that obesity decreases the activity of major cardiac voltage-gated K^+^ channels for impaired cardiac repolarization was tested. I_peak_ (Fig 3A) currents were recorded. Our data suggested that, in addition to the test potential of –40 mV, outward K^+^ currents were smaller in the myocytes of HFD mice than those in control cells at all other test potentials (Fig 3B). Compared with the I_peak_ current density in control mice (14.0±0.9 pA/pF), the I_peak_ density at +70 mV was reduced in HFD mice (10.6±0.5 pA/pF; *p*<0.01) (Fig 3C).

To confirm the I_to_ current, a pharmacological approach of 4-AP was utilized (Fig 3D). Representative I_to_ values recorded from the ventricular myocytes of control and HFD mice are shown in Fig 3D. The I_to_ current corresponds to phase 1 of the AP, and the I_to_ current density increased with increasing test potential. I_to_ current density was smaller in HFD mice (6.1±0.5 pA/pF) than in controls (9.4±0.6 pA/pF; *p*<0.01) (Fig 3E and 3F). The decrease in current density was attributed to changes in channel gating kinetics or protein expression. Given this association, the gating mechanism of I_to_ in the obesity phenotype were investigated. I_to_ voltage dependence of the steady-state inactivation (SSI) curve in HFD mice was found to shift to the left (Fig 3H). V_1/2, inact_ was slightly increased from –46.4±1.0 mV to –51.5±1.2 mV, indicating that the I_to_ current is more prone to inactivation with obesity. However, the voltage dependence of steady-state activation (SSA) curve as well as the recovery from inactivation (RFI) curve were unaltered (Fig 3G and 3I). The 4-AP-sensitive I_K,slow_ showed negligible reduction in HFD mice (*p*>0.05) (Fig 3J and 3K). Analysis of the decay phases of outward currents evoked during 300ms depolarization revealed that the current decay was well described by the sum of two exponentials, fast and slow decay time constants respectively, as described under “Supplemental Methods” (S1 File). It seems fast and slow decay time constants is slower in HFD cardiomyocytes at all test potentials, for example, at 40 mV the slow decay time constant from control myocytes was 1030.1±23.5 ms compared with 1077.9±10.8 ms for HFD group (*p*>0.05), the fast decay time constant from control myocytes was 71.2±5.4 ms compared with 85.9±8.3 ms for the HFD group (*p*>0.05), however the difference was not statistically significant (Fig 3L).

### Inhibition of K^+^ channel proteins in HFD mice ventricles

The proteins that constitute the repolarization-related potassium ion channels in mouse include K_V_4.2, KChIP2, and K_V_1.5. I_to_ is composed of α (K_V_4.2) and β (KChIP2) subunits that modulate gating and/or trafficking of channels to the cell membrane. Western blotting of the ventricle (Fig 3M) revealed significant reductions in K_V_4.2 and KChIP2 levels in the HFD group (*p*<0.01 and *p*<0.05; Fig 3N and 3O). Moreover, the analysis demonstrated a reduction in K_V_1.5 which constitute I_K,slow_ (*p*<0.05; Fig 3P). These data indicate that the decrease in outward potassium current may be mainly due to the inhibition of protein expression after obesity, especially the K_V_4.2 and KChIP2 proteins that make up the I_to_ current.

### Unaltered I_K1_ current and K_ir2.1_ protein in ventricles of HFD mice

**Fig 4:**
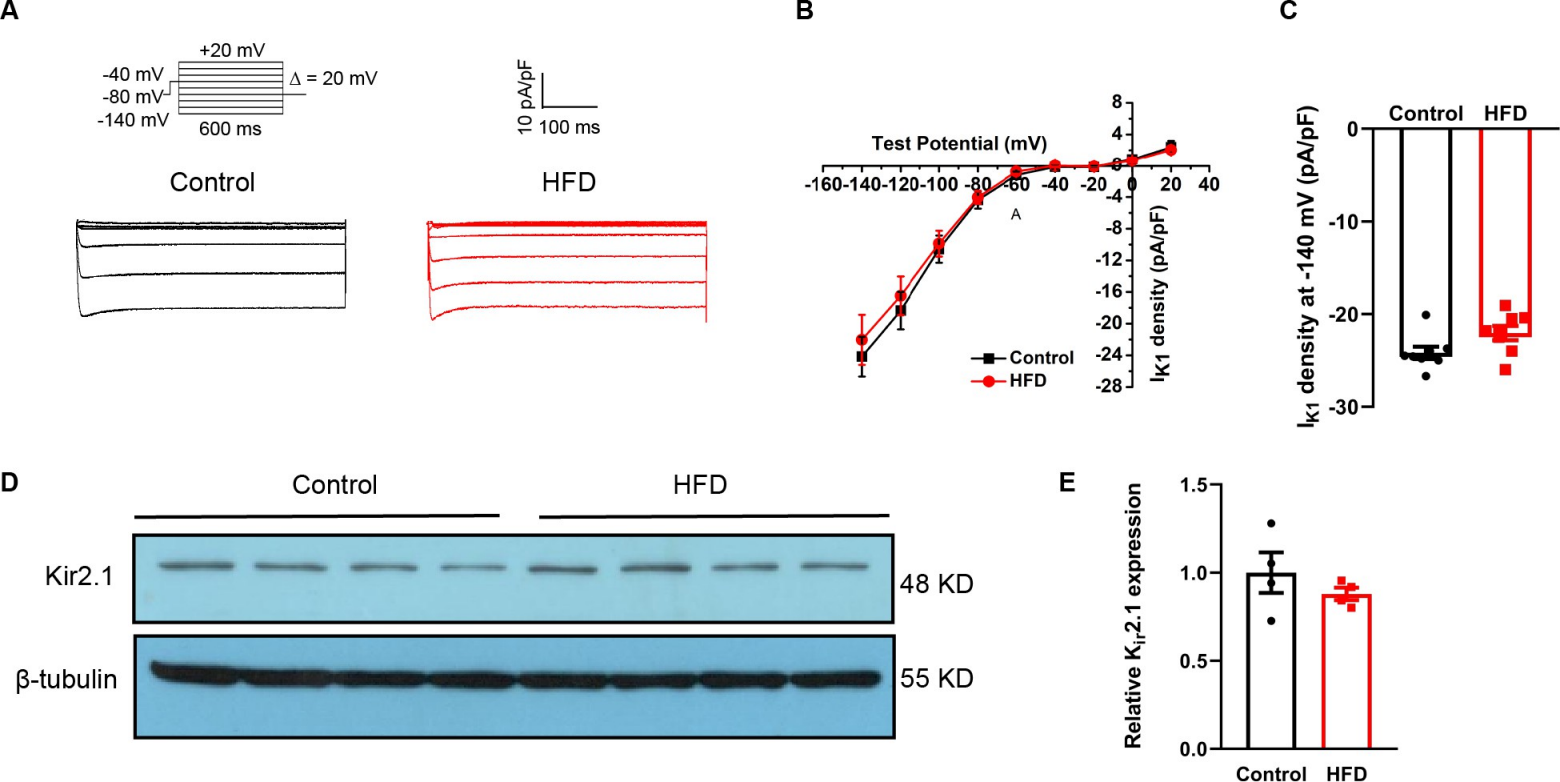
The consequences of an HFD were not attributed to inward-rectifier K+ current. A: Representative I_K1_ in the two groups. B: Voltage relationship (I-V curves) in the two groups. C: I_K1_ at –140 mV in the two groups. D: Representative Western blots of K_ir_2.1. E: Quantification of K_ir_2.1. for A-C, n = 8 cells; three hearts per group. for D-E, n = 4 animals per group.

I_K1_ corresponds to phase 4 of the AP and resting membrane potential. Representative original I_K1_ in the two groups are shown in Fig 4A. Analysis showed a non-significant reduction in I_K1_ densities at –140 mV (*p*>0.05) (Fig 4B and 4C). No significant difference was present in K_ir_2.1 levels (*p*>0.05; Fig 4D and 4E).

### Increased ventricular oxidative stress in HFD mice and generation of Nox2-knockout mice

**Fig 5:**
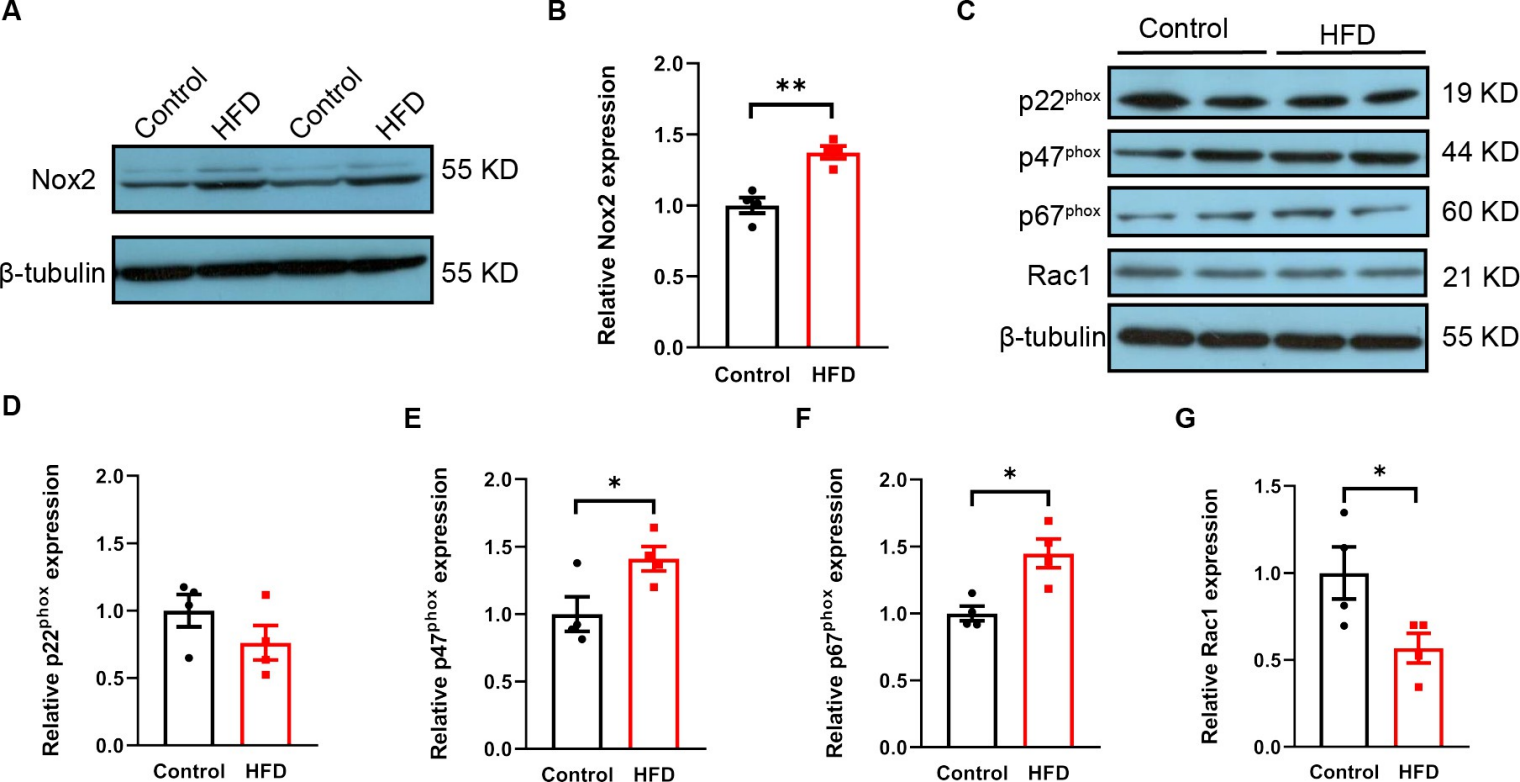
HFD causes activation of Nox2. A: Representative Western blots of Nox2 in control and HFD mice. B: Quantification of Nox2. C: Representative Western blots of p22^phox^, p47^phox^, p67^phox^, and Rac1. D: Quantification of p22^phox^. E: Quantification of p47^phox^. F: Quantification of p67^phox^. G: Quantification of Rac1. n = 4 animals per group. *p<0.05, **p<0.01. HFD, high-fat diet.

Obesity and cardiac repolarization have been independently linked to oxidative stress, but insufficient evidence currently exists to establish a direct link between obesity and ventricular oxidation. Our study found that Nox2 protein levels in ventricular tissue were increased in the HFD mice (*p*<0.01) (Fig 5A and 5B), but the association between Nox2 subunits and obesity remains unclear. The analysis of p47phox and p67phox subunits demonstrated a statistically significant increase in the HFD group (*p*<0.05, Fig 5C, 5E and 5F), while p22phox expression showed no change (*p*>0.05, Fig 5D). Rac1, on the other hand, was decreased in the HFD group (*p*<0.05, Fig 5G).

### Deletion of Nox2 rescues I_peak_ and I_to_ currents

**Fig 6:**
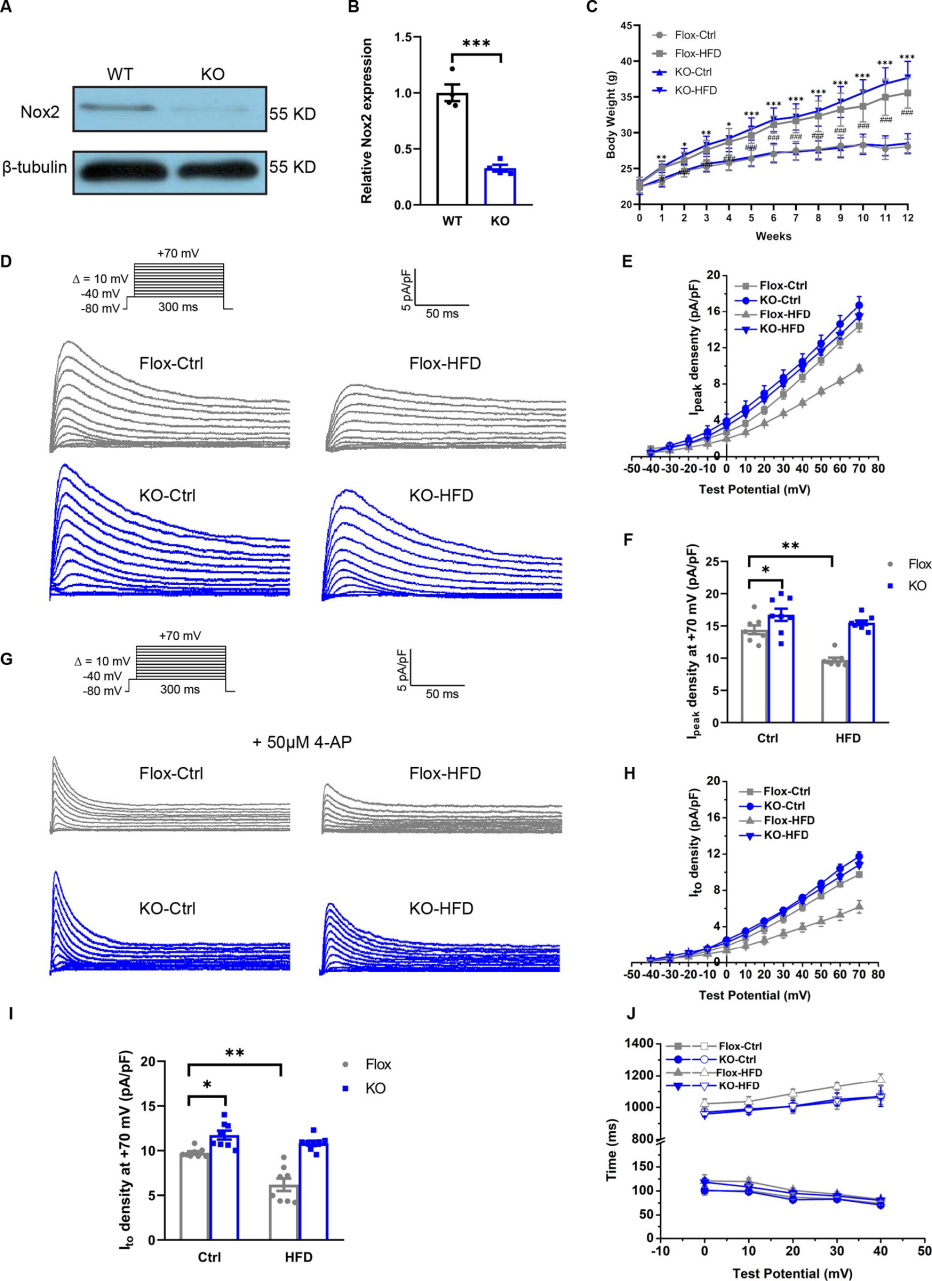
Genetic ablation of Nox2-rescued I_peak_ and I_to_ inhibited by HFD. A: Representative Western blots of Nox2 in KO and WT mice. B: Quantification of Nox2 in KO and WT mice. C: Body weights in Flox and KO mice. D: Representative Ipeak recorded from Flox (grey) and KO (blue) ventricular myocytes of mice from the four groups. E: Voltage relationship (I-V curves) in the four groups. F: Ipeak at +70 mV in the four groups. G: Representative Ito recorded from Flox (grey) and KO (blue) ventricular myocytes of mice from the four groups. H: Voltage relationship (I-V curves) in the four groups. I: Ito at +70 mV in the four groups. J: Analyzed fast (lower series) and slow (upper series) inactivation time constants (τ values) from four groups. For A and B, n = 4 animals per group; for C, n = 12 animals per group; for D-J, n = 8 cells; three or four hearts per group. *p<0.05, **p<0.01, ***p<0.001 vs. KO-Ctrl; #*p*<0.05, ###*p*<0.001 vs. Flox-Ctrl. HFD, high-fat diet; KO, knockout.

To further investigate the effects of Nox2 deletion, we generated *Nox2*^*fl/fl*^ αMyHC-Cre (Nox2-knockout [KO]) mice using αMyHC-Cre, which is specific to mouse hearts. The analysis of KO mice is shown in Fig 6A and 6B. The body masses of Flox and KO mice are shown in Fig 6C. ECG recordings were conducted on Nox2-KO mice and littermate Flox mice (Table 2). Interestingly, Nox2-KO mice did not exhibit prolonged QT and QTc intervals in the HFD group, indicating that cardiac-specific Nox2-KO is protective in vivo. These data suggest that impaired obesity-mediated repolarization is partly attributed to Nox2.

**Table 2:** ECG characteristics of Flox, and KO mice before and after obesity.

ECG parameters	Control group		HFD group	
	Flox	KO	Flox	KO
R-R (ms)	133.1±4.7	129.6±2.4	139.3±3.1	130.0±4.3
QRS (ms)	12.0±0.5	11.7±0.6	12.0±0.3	11.7±0.5
QT (ms)	53.0±0.8	52.0±1.8	62.7±1.5**	53.3±1.6
QTc (ms)	46.0±0.9	45.7±1.5	53.1±0.9**	46.8±1.3

n = 7–8 animals per group. Data are presented as mean ± SEM. ECG, electrocardiogram; KO, knockout; HFD, high-fat diet

**p<0.01 vs. correspondent control diet group.

Representative I_peak_ values recorded from Flox (gray) and KO (blue) ventricular myocytes of mice are shown in Fig 6D. First, KO-Ctrl mice displayed an increase in I_peak_ current under normal diet when compared with Flox-Ctrl mice (16.7±0.9 pA/pF vs 14.4±0.6 pA/pF, *p*<0.05, Fig 6E and 6F), indicating that KO can increase potassium current density. Furthermore, the rise in I_to_ current accounts for nearly all of the increased I_peak_ current (KO-Ctrl 11.8±0.5 pA/pF vs Flox-Ctrl 9.7±0.1 pA/pF, *p*<0.05, Fig 6G). Secondly, in accordance with the differences in ventricular myocytes of wild-type mice, decreased I_peak_ and I_to_ (Fig 6H and 6I) densities were observed in Flox-HFD mice compared with Flox-Ctrl mice (*p*<0.01).Third, the I_peak_ current density after KO-HFD demonstrated a non-statistically significant reduction compared to KO-Ctrl mice (Fig 6F), indicating that KO partly reversed the decrease in potassium current induced by the high-fat diet compared to the changes in Flox mice after obesity. These data indicate that obesity can reduce I_peak_ and I_to_ current density through the regulatory mechanism of Nox2.

Fig 6J shows the data of inactivation kinetics, at 40 mV the slow decay time constant from Flox-Ctrl, KO-Ctrl, Flox-HFD and KO-HFD myocytes was 1061.3±41.7 ms, 1072.1±65.3 ms, 1172.7±39.7 and 1068.1±12.9 ms, respectively. But there was no significant change in the inactivation time constants of the four groups at all test potentials, indicating that both high-fat and KO have no significant effect on the inactivation kinetics of outward potassium current.

## Discussion

Despite the increasing prevalence of obesity, its contribution to the pathophysiology of ventricular arrhythmias remains unclear. Our study emphasizes the importance of obesity in acquired LQTS. Our results revealed the electrophysiological mechanisms underlying the contribution of obesity to the electrical remodeling associated with LQTS or impaired cardiac repolarization. We further highlighted the role of the modified K^+^ channel in the electrical remodeling observed in HFD mice.

After 12 weeks of control or high-fat feeding, we found that the endocardium, myocardial membrane, and epicardial structures of the heart tissue of both the control and HFD mice groups were clear, and there were no significant abnormalities in the heart wall and chamber. HFD mice showed loose arrangement of myocardial cells, widened intercellular spaces, and increased cardiomyocyte cross-sectional area. The results of Masson staining showed that there was no statistically significant difference in the degree of fibrosis between the two groups, which is different from previous reports. Wang et al. [21] and Pan et al. [22] reported an increase in fibrosis in mice after high-fat feeding, but the difference lies in the duration of modeling. We analyzed that the reason may be due to the different duration of special diet feeding and age variations of the mice from which the hearts were harvested. Similar to other reports in mouse, rabbit, and rat models [12, 23–25], surface ECGs exhibited prolonged QT intervals in HFD mice. Numerous studies have demonstrated a link between obesity and ventricular oxidation [11, 13], and recent reports have demonstrated that Nox2 is correlated with impaired cardiac repolarization. Joseph et al. [12] reported that Nox2 activity in the ventricular tissue was increased by an HFD and could be reversed by the Nox inhibitor apocynin. With an HFD, Nox2-KO mice did not exhibit prolonged QT and QTc intervals, demonstrating that Nox2-KO is protective in vivo. These data suggest that impaired obesity-mediated repolarization is partly attributed to ventricular oxidation.

An HFD increases APD [9, 26]. Ashrafi et al. [9] demonstrated that an HFD increases APD in rats, while Torres-Jacome et al. [10] showed that obese rats with metabolic syndrome had increased APD in the ventricles. The APD data in our study matched the in vitro results previously published for the murine heart [27]. Our study showed that HFD mice had prolonged ventricular myocyte APD, especially in terms of APD_20_ and APD_90_. APD_20_ corresponded to phase 1 of the AP. The K^+^ channels were transiently opened, causing an outward K^+^ current. This resulted in a period of rapid repolarization. APD_90_ corresponded to phases 3 and 4 of the AP, which resulted in a period of late repolarization. Therefore, QT interval prolongation may be attributed to abnormalities in ventricular repolarization and APD.

K^+^ currents play an important role in lipid toxicity models and impaired cardiac repolarization. Compared with those from wild-type mice, ventricular cells derived from MHC-PPARα mice showed increased I_K1_ density and decreased I_to_ and I_K,slow_ densities [27, 28]. Recently, Torres-Jacome et al. [10] recorded K^+^ currents in isolated myocytes and showed a decrease in the I_to_ current amplitude in rats with metabolic syndrome. In agreement with the aforementioned study, amplitudes of I_peak_ and I_to_ currents were reduced in HFD mice compared with those of control mice based on our direct electrophysiology testing.

Most cases of LQTS related to K^+^ channels include defects in channel gating [29]. The channel gating of I_to_ includes SSA, SSI, and RFI curves. Here, rapid activation, inactivation, and recovery from inactivation were shown in I_to_. Moreover, SSI in HFD mice was slightly shifted to the left, indicating a tendency toward inactivation after obesity. Our findings on inactivation kinetics show that the fast and slow inactivation time constants of the high-fat group appear to be longer, but there is no statistical difference. This may be due to our study of the entire ventricle, which differs from previous studies which have reported differences in cell inactivation constants from the right and left ventricles, as well as differences in inactivation constants of apex, base, endo and epi in the left ventricle. For example, Brunet reports that the tau decay value of I_K,slow_ in left ventricular endo cells (1441 ± 40 ms) is significantly greater than that in left ventricular Epi cells (1191 ± 42 ms) [30]. Our single-cell records did not differentiate ventricular myocytes from different locations, which may have masked the differences in inactivation constants. We are interested in exploring this content in the future.

Our results revealed a significant reduction in K_V_4.2 and KChIP2 protein levels in obese mice compared with those in controls. I_to_ consists of pore-forming α subunits and accessory β subunits, modulating channel gating [29, 31]. In mice, I_to_ channels reflect the heteromeric assemblies of K_V_4.2, K_V_4.3, and KChIP2 [32]. Huang et al. [8] demonstrated a prolonged QT interval in HFD-induced obese mice. Reduced K_V_1.5 protein levels were also consistently found in our study. However, it should be noted that in contrast to higher animals, such as rabbits, canines, and humans, K_V_1.5—found only in the atrium in mice—is not required for ventricular repolarization [32]. Moreover, K_V_4.3 is not part of functional I_to_ in mice [33].

Our data demonstrated that Nox2 protein expression in the ventricular tissue was increased. Further analysis of the p47phox and p67phox subunits demonstrated a statistically significant increase in HFD mice, which partly accounted for the increased NADPH oxidase activity. Rac1, however, was decreased in HFD mice. Two membrane-associated subunits (gp91phox [Nox2] and p22phox), three cytosolic subunits (p47phox, p67phox, and p40phox), and the G-protein Rac are known to be found in the classic NADPH oxidase structure. The multicomponent complex remains inactive and physically dissociated. To activate Nox2, complex protein-protein interactions and translocation from the cytoplasm to the cell membrane are required. The transfer of p47phox and p67phox subunits is necessary for the formation of active Nox2 enzyme complexes. Rac1 is a small GTPase, and its activation binds to p67phox, triggering Rac1-p67phox dimer translocation from the cytoplasm to the cell membrane. Our study showed decreased Rac1 in the HFD group, including those in the membrane and cytosol. The cell membrane-to-cytoplasm ratio indicates the activation of the complex. However, data on this ratio is lacking in our study. Despite these results, whether and how these subunits influence Nox2 function in obesity is poorly understood and requires further investigation.

Recent reports have demonstrated that Nox2 modulates K^+^ channels [17, 18]. Given this association, the role of Nox2 expression in regulating cardiac K^+^ channels in obesity was determined. Our results revealed that Nox2 mediates the effects of an HFD on I_peak_ and I_to_. In response to the HFD, I_peak_ and I_to_ increased in KO-HFD mice. Moreover, the reductions in both outward K^+^ currents were prevented in KO-HFD cardiomyocytes, which had similar I_peak_ and I_to_ current densities to the normal diet groups. These data suggest that Nox2 partly normalizes HFD-induced outward K^+^ currents.

Collectively, these findings support our hypotheses. We showed that I_peak_ and I_to_ were significantly reduced in HFD mice. Substantial differences in the I_peak_ and I_to_ current densities account for the broader APD and impaired cardiac repolarization. Conditional cardiac-specific deletion of Nox2 reduced ventricular oxidative stress and partially normalized voltage-gated K^+^ channel activity. We determined that HFD mice were not only more prone to developing prolonged cardiac repolarization, but the adverse effects of Nox2 acted as a partial mediator of this risk.

Our study had some limitations. Studies have suggested a cross-talk between Nox and ROS generated by mitochondria. Cross-talk can also be initiated in the mitochondria by conditions such as an HFD [34–37]. Whether and how cross-talk influences Nox2 function in obesity is poorly understood and requires further investigation. In the study, only male mices were used. However, as is well known, females have longer QTc intervals than males [38]. As early as 1978, Hashiba K first observed that females were the more predominant LQTS patients [39]. Therefore, including females is crucial. However, in this experiment, we aimed to evaluate the effect and mechanism of obesity on repolarization delay. In the pilot study, HFD was also used, but females did not reach the weight threshold. Secondly, in studies on mice, it was found that using HFD resulted in significantly greater weight gain in male compared with females [19]. It is indeed worthwhile to analyze the obesity related repolarization delay in females by improving the preparation of obesity models in the future.

In conclusion, abnormalities in repolarization and obesity are associated with oxidative stress and exacerbate ventricular electrical remodeling. Our study emphasizes the importance of obesity in acquired LQTS and revealed the electrophysiological mechanisms underlying the contribution of obesity to electrical remodeling that causes impaired cardiac repolarization. Cardiac-specific ablation of Nox2 can alleviate the HFD-related decreased expression of outward K^+^ channels. This study clarified the mechanisms underlying the pathogenic consequences of obesity-related repolarization abnormalities, the contribution of oxidative stress, and electrophysiological alterations.

## Supporting information

S1 Fig The original uncropped blot images. (PDF)

S1 File Supplemental material. (DOCX)
