# Supplementary material for: Obesity-induced activation of NADPH oxidase 2 prolongs cardiac repolarization via inhibiting K+ currents
Source: PLoS One. 2024 Dec 31;19(12):e0316701. doi: 10.1371/journal.pone.0316701 (PMC11687869; doi:10.1371/journal.pone.0316701)
Supplement: S1 Fig — (PDF) [file pone.0316701.s001.pdf]

The full uncropped of western blot image

For Fig 3M

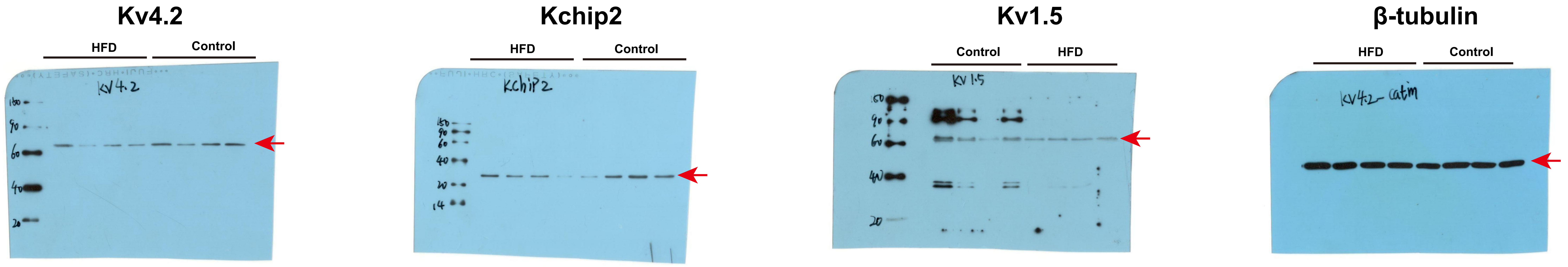

For Fig 4D

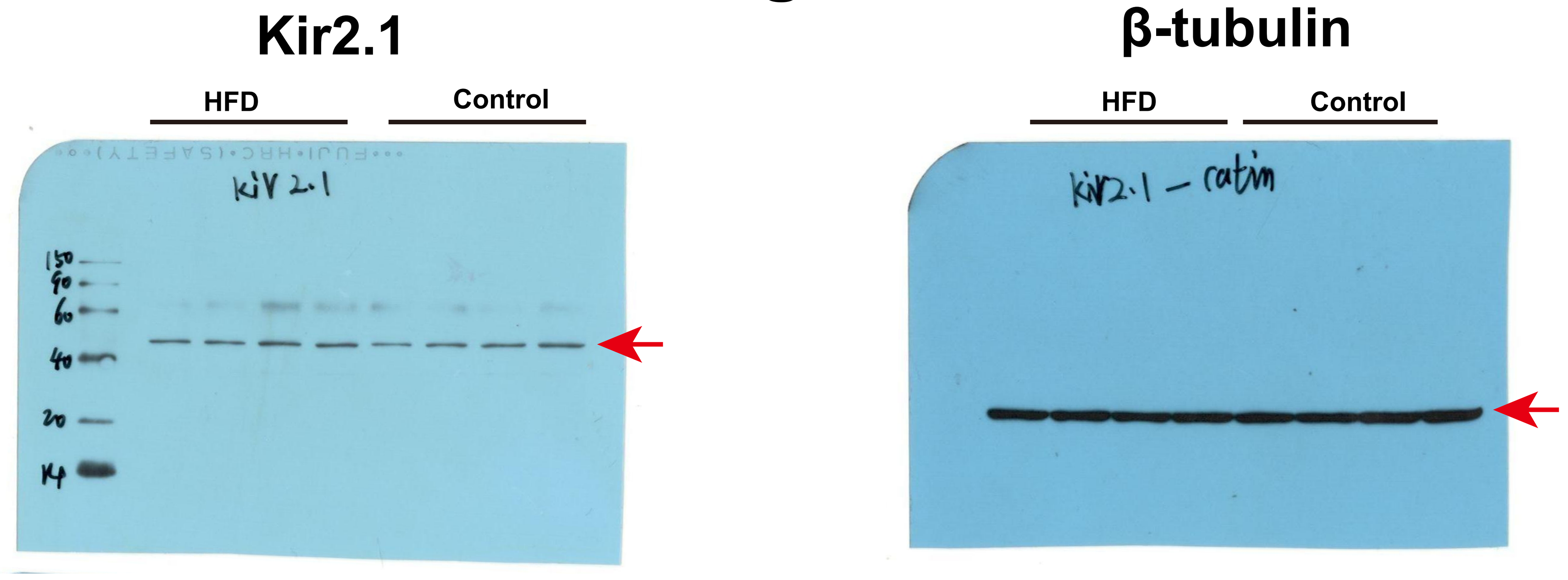

For Fig 5A

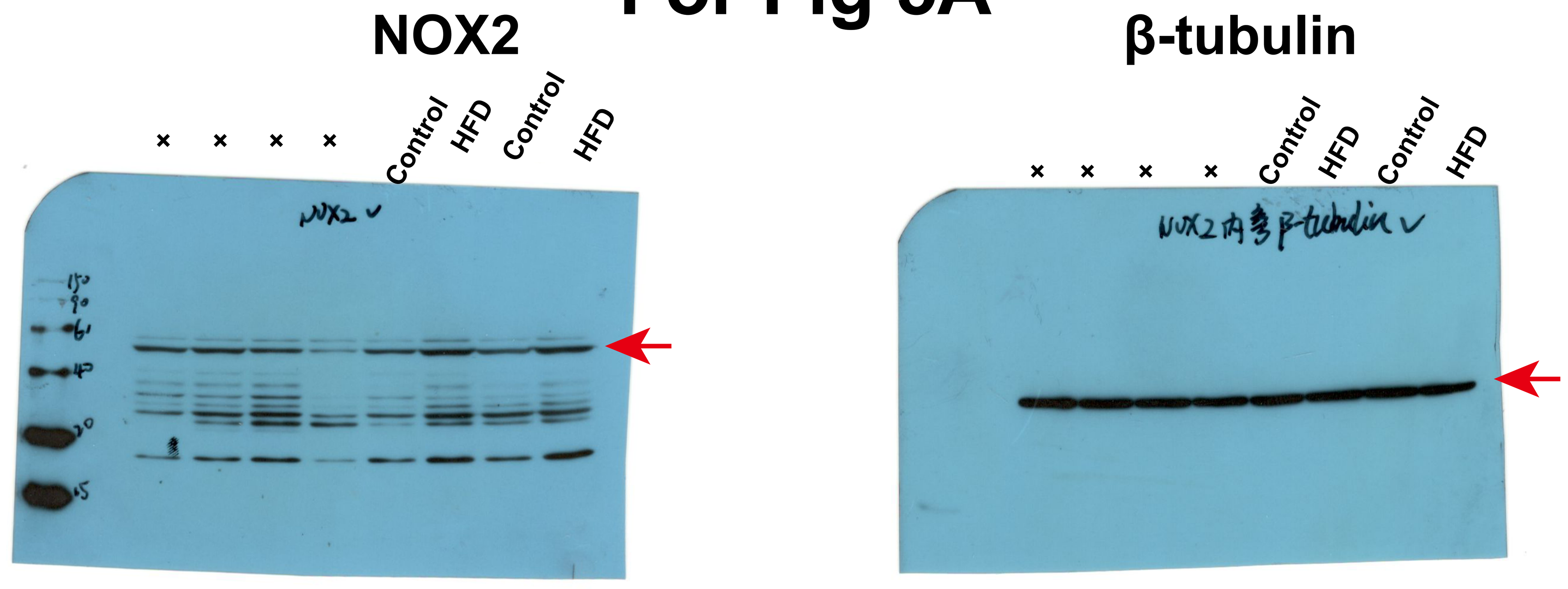

For Fig 5C

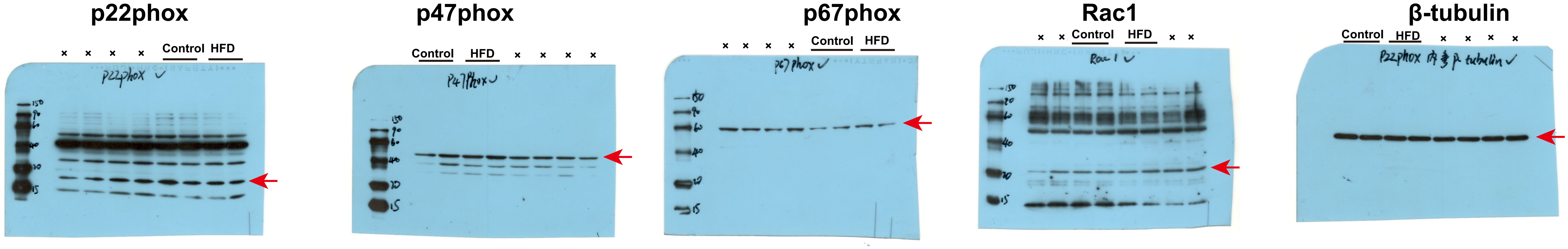

For Fig 6A

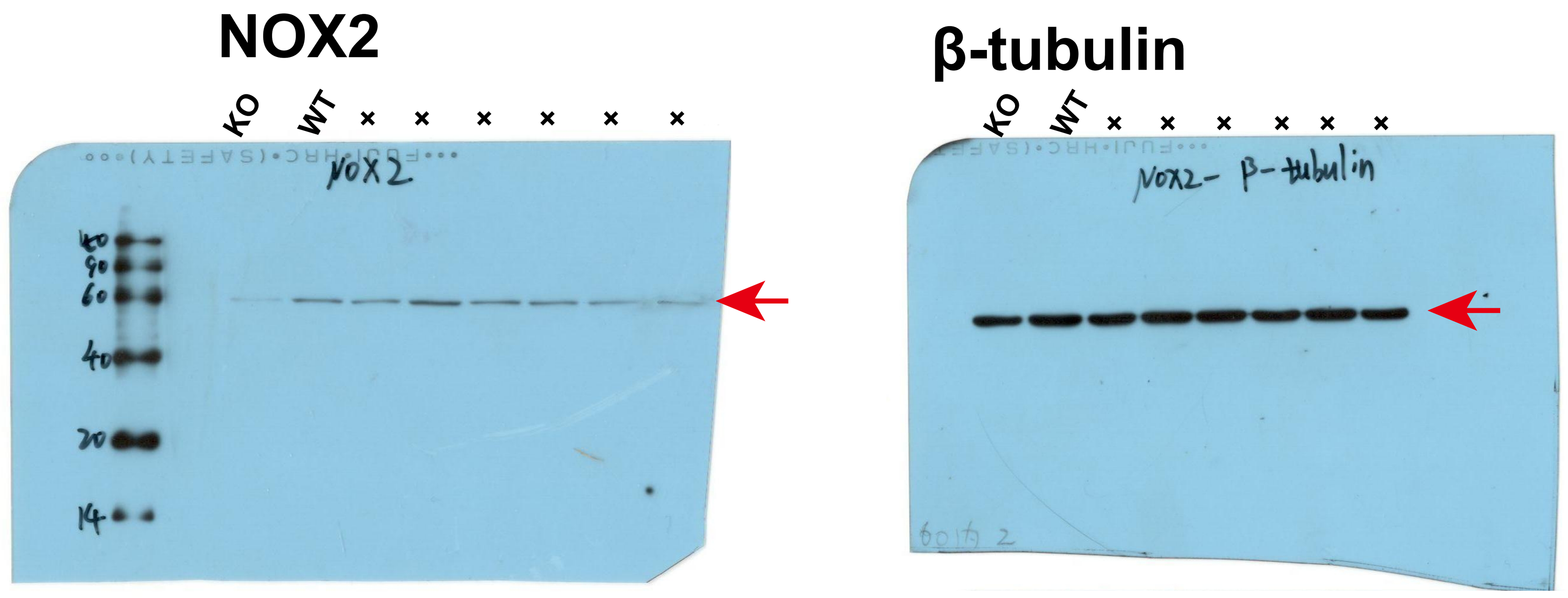

← Cropped region

× Lanes not included in the final figure
