# Supplementary material for: Obesity-induced activation of NADPH oxidase 2 prolongs cardiac repolarization via inhibiting K+ currents
Source: PLoS One. 2024 Dec 31;19(12):e0316701. doi: 10.1371/journal.pone.0316701 (PMC11687869; doi:10.1371/journal.pone.0316701)
Supplement: S1 File — (DOCX) [file pone.0316701.s002.docx]

SUPPLEMENTAL MATERIAL

# Supplemental Methods

## Generation of KO mice

Floxed Nox2 (*Nox2^fl/fl^*) and KO mice with a C57/BL6J genetic background were generated using CRISPR/Cas genome engineering. The selection of exon 5 to insert the Floxed allele led to the deletion of this region, causing a loss of function of the mouse Cybb gene. By crossing Flox mice with αMyHC-Cre mice, cardiac-specific KO mice (*Nox2^fl/fl^* αMyHC-Cre mice) and littermate controls (Flox mice) were produced. Male KO and Flox mice were fed a high-fat diet (HFD) or a normal diet for 12 weeks.

## Whole-cell patch-clamp recordings

Following heparin (125 U) injection, each heart was removed. After perfusing Ca^2+^-free Tyrode buffer gassed with O_2_ for 2 min, a collagenase II (Worthington) and protease mixture was infused into the coronary arteries. To extract single cells, the left ventricular (LV) tissue was gently divided using tiny forceps and pipetted for a short time. Ca^2+^, including BSA, was elevated over three steps to 0.5 mM. The suspension contained 65%–80% rod-shaped myocytes. The Ca^2+^-free Tyrode buffer contained 112.9 mM NaCl, 4.7 mM KCl, 1.2 mM KH_2_PO4, 1.1 mM Na_2_HPO4, 1.2 mM MgSO4, 11.9 mM NaHCO3, 10.0 mM KHCO3, 10 mM HEPES, 5.5 mM Glucose and 14.9 mM Taurine.

A solution containing individual non-contracting rod-shaped cardiomyocytes was dispersed into chambers set atop an inverted microscope's stage (Olympus). The external solution was continually superfused into the cardiomyocytes once they had settled to the bottom, which took 5–10 min.

Generally, the series resistance was 2–3 MΩ, with an 80% compensation. The trials were conducted at room temperature. A voltage clamp was used to measure K^+^ currents. Current amplitudes were divided by myocyte membrane capacitance to calculate current densities.

In the majority of the cells dispersed from adult mouse ventricles, three distinct K^+^ current constituents were identified: I_to,f_, I_K,slow_, and I_ss_. Proper protocols were used to separate K^+^ current components, and proper drugs were used to inhibit mixed Na^+^ and Ca^2+^ currents.

To distinguish Ito from I_K,slow_, 50μM 4-aminopyridine (4-AP) was added to the mixture. Furthermore, 0.1 mM CdCl_2_ was used to inhibit the Ca^2+^ current, and 10 μM tetrodotoxin (TTX) and 25 ms of depolarization to –40 mV were used to block the inward Na^+^ current. The gating kinetics of I_to_ in the obesity phenotype were investigated. The steady-state activation (SSA) kinetics of I_to_ were recorded using the same protocol as that used to determine the current-voltage relationships. To fit SSA, the Boltzmann equation (1) was used, as follows:

G/Gmax=1/(1+exp[(Vm–V1/2)/k]) (1)

To ascertain the steady-state inactivation (SSI) of I_to_, a protocol consisting of 1000 ms of conditioning pulses between –70 mV and +20 mV, succeeded by a test pulse of +50 mV for 300 ms, was employed. To fit SSI, the Boltzmann equation (2) was used, as follows:

I/Imax=1/(1+exp[(V1/2–Vm)/k]) (2)

A protocol of twin pulses was employed to assess recovery from inactivation (RFI), as described previously [1].

The fast and slow decay phases of the currents were fitted by the sum of two exponentials (3) as follows [1,2]:

y(t)= A_1_ * exp(-t/τ1) +A_2_ * exp(-t/τ2) +A_ss_ (3)

I_K1_ was sensitive to 300 μM Ba^2+^. A protocol of 600 ms of test pulses ranging from −140 to +20 mV was used to measure I_K1_. To record APs, TTX and CdCl_2_ were omitted. APs were recorded after injections of a depolarizing current at a frequency of 1 Hz.

For IK recording, the external solution in the patch electrode contained 140 NaCl, 1 CaCl_2_, 1 MgCl_2_, 4 KCl, 10 HEPES, and 5 mM glucose, with the pH adjusted to 7.3–7.4 with NaOH. The internal solution contained 130 KCl, 1 MgCl_2_, 5 EGTA, 10 HEPES, 10 glucose, and 5 mM Na_2_ATP, with the pH adjusted to 7.2–7.3 with KOH.

For AP recording, the external solution in the patch electrode contained 140 mM NaCl, 1 mM CaCl_2_, 1 mM MgCl_2_, 10 mM HEPES, 5 mM glucose, and 4 mM KCl, with the pH adjusted to 7.35 with CsOH. The internal solution contained 120 mM K-aspartate, 20 mM KCl, 1 mM MgCl_2_, 4 mM Na_2_ATP, 10 mM HEPES, 10 mM glucose, and 0.1 mM GTP, with the pH adjusted to 7.25–7.35 with CsOH.

**Supplemental References**

1. Xu H, Guo W, Nerbonne JM. Four kinetically distinct depolarization-activated k+ currents in adult mouse ventricular myocytes. J Gen Physiol 1999;113: 661-78. doi.org/10.1085/jgp.113.5.661.

2. Xu H, Barry DM, Li H, Brunet S, Guo W, Nerbonne JM. Attenuation of the slow component of delayed rectification, action potential prolongation, and triggered activity in mice expressing a dominant-negative kv2 alpha subunit. Circ Res 1999;85: 623-33. doi.org/10.1161/01.res.85.7.623.
